# Metformin Ameliorates Synaptic Defects in a Mouse Model of AD by Inhibiting Cdk5 Activity

**DOI:** 10.3389/fncel.2020.00170

**Published:** 2020-06-24

**Authors:** YaLi Wang, JianHua Zhao, Fang-Li Guo, XiaHuan Gao, Xine Xie, ShouQing Liu, Xin Yang, XinFeng Yang, LuYi Zhang, YuXiao Ye, LiBing Fan, JianGang Wang

**Affiliations:** ^1^Key Laboratory for the Brain Research of Henan Province, Department of Physiology and Neurobiology, Xinxiang Medical University, Xinxiang, China; ^2^Henan Key Laboratory of Neurorestoratology, The First Affiliated Hospital of Xinxiang Medical University, Xinxiang, China; ^3^Department of Neurology, Anyang District Hospital of Puyang City, Anyang, China; ^4^Department of Pathology, People’s Hospital of Tongchuan, Tongchuan, China; ^5^Department of Neurology, The Second Hospital of Jinhua, Jinhua, China; ^6^Department of Physiology, School of Basic Medicine and Tongji Medical College, Huazhong University of Science and Technology, Wuhan, China; ^7^Department of Pathophysiology, Xinxiang Medical University, Xinxiang, China; ^8^Henan Key Laboratory of Biological Psychiatry, The Second Affiliated Hospital of Xinxiang Medical University, Xinxiang Medical University, Xinxiang, China

**Keywords:** AD, Cdk5, p35, p25, metformin, synapse

## Abstract

Cyclin-dependent kinase 5 (Cdk5) is a serine/threonine kinase that is activated by the neuron-specific activators p35/p39 and plays important roles in neuronal development, synaptic plasticity, and cognitive behavior. However, the proteolytic cleavage of p35 to p25 leads to prolonged and aberrant Cdk5 activation and results in synaptic depression, highly mimicking the early pathology of Alzheimer’s disease (AD). Therefore, Cdk5 inhibition is a potential promising strategy for AD drug development. Here in the present study, we showed that metformin, the most widely used drug for type 2 diabetes, suppressed Cdk5 hyper-activation and Cdk5-dependent tau hyper-phosphorylation in the *APP/PS1* mouse hippocampus. We also identified the underlying molecular and cellular mechanism that metformin prevented Cdk5 hyper-activation by inhibiting the calpain-dependent cleavage of p35 into p25. Moreover, chronic metformin treatment rescued the core phenotypes in *APP/PS1* mice as evidenced by restored spine density, surface GluA1 trafficking, Long-term potentiation (LTP) expression, and spatial memory. Altogether our study discovered an unidentified role of metformin in suppressing Cdk5 hyper-activation and thus preventing AD pathogenesis and suggested that metformin is a potential promising AD therapeutic drug.

## Introduction

Alzheimer’s disease (AD) is a progressive neurodegenerative disease characterized by synaptic depression, synapse loss, and cognitive impairment. It has become the sixth leading cause of death and is a global threat to public health. This disease afflicts ~44 million people worldwide and accounts for ~605 billion in medical expenses in 2019. However, the patient number and the medical costs are still going up and are expected to double by 2050 (data from the Alzheimer’s association) due to the lack of an effective cure for AD, highlighting the importance and urgency of developing new drugs and therapies. Unfortunately, many drugs that target amyloid beta (Aβ) deposits failed in preclinical or clinical trials. All these facts drove us to look carefully into the pathophysiological mechanisms underlying the synaptic failures in AD and hope to find out some promising targets for drug development.

Among different signaling molecules, cyclin-dependent kinase 5 (Cdk5) is a potential therapeutic target. Cdk5 is a proline-directed serine/threonine kinase and is activated by its neuron-specific activators, p35 or p39. Cdk5 plays indispensable roles in the early stage of neural development including neuronal migration, neurite development, hippocampus neurogenesis, and synaptic formation and elimination (Dhavan and Tsai, 2001; Su and Tsai, 2011). However, the aberrant activation of Cdk5 is responsible for the pathogenesis of several neurodegenerative diseases and psychiatric disorders including AD, Parkinson’s disease (PD), and depression (Cheung and Ip, 2012; Cortés et al., 2019). Indeed it is reported that the proteolytic cleavage of p35 produces p25, causing a prolonged and aberrant Cdk5 activation when the neurons are exposed to cellular stresses such as neurotoxicity and Aβ deposits (Shukla et al., 2012). For example, p25 is found to be accumulated in the brains of AD patients (Patrick et al., 1999). In addition, another recent study showed that chronic p25 production induced initially concurrent synapse density reduction and synaptic size increase and later persistent synapse elimination in intact brains, highly recapitulating the early Alzheimer’s synaptic pathology (Sheng et al., 2016). Thus, it has been proposed that Cdk5 activity inhibition can be a potential promising therapeutic strategy for AD treatment. Indeed many studies have shown that the pharmacological or the genetic inhibition of Cdk5 activity can prevent synaptic loss and neuronal death and exert some protective effect in mouse models of AD and PD (Lau and Ahlijanian, 2003; Piedrahita et al., 2010; Liu et al., 2016; Seo et al., 2017; He et al., 2018). However, preclinical or clinical trials with some Cdk5 inhibitors were not promising due to severe side effects or off-target effects (Cicenas et al., 2015). This prompted us to re-search the library of US Food and Drug Administration (FDA)-approved drug to find out some potential Cdk5 inhibitors and examine if they could be promptly repurposed as AD therapeutic.

Accumulating evidence has suggested that type 2 diabetes mellitus (T2DM) and AD share a similar pathophysiology. It has even been hypothesized that AD might be “type 3 diabetes” (de la Monte, 2014). For example, several clinical studies showed that diabetes patients had increased AD risk by two- to threefold. Meanwhile, more than 80% of AD patients showed abnormal blood glucose level and developed diabetes, indicating a high correlation between the onset of T2DM and AD (Janson et al., 2004). In several recent studies, scientists reported that one family of anti-diabetic drugs, the thiazolidinediones (TZD) drugs troglitazone and pioglitazone, could inhibit Cdk5 kinase activity and ameliorate some synaptic deficits in mouse models of AD (Cho et al., 2013; Chen et al., 2015). However, the application of TZDs is often associated with edema, weight gain, macular edema, and heart failure (Rizos et al., 2009), which prevent their wide use in AD patients. In contrast, metformin, the most widely used FDA-approved drug for type 2 diabetes and whose long-term safety and tolerability have been well studied and documented, is one promising compound for several reasons: (1) metformin treatment exerts some protective and beneficial effect in neurotoxicity by decreasing intracellular calcium influx (Jang and Park, 2018); (2) metformin administration corrects synaptic failures and cognitive deficits in a *Drosophila melanogaster* fragile X model and a mouse model of fragile X syndrome by normalizing ERK signaling (Gantois et al., 2017; Monyak et al., 2017), whose hyper-activation also contributes to AD pathology (Sun and Nan, 2017); and (3) metformin treatment reduces endometrial cancer size by reducing the Cdk5-dependent phosphorylation of STAT3 at serine 727 (Leidgens et al., 2017). Based on these reports and our preliminary data, we proposed to examine whether metformin treatment can rescue synaptic dysfunctions and improve cognitive functions in AD mouse models by inhibiting Cdk5 kinase activity.

## Materials and Methods

### Animals

The *APP/PS1* (APPswe + PSEN1dE9) transgenic mice were purchased from Jackson Laboratory. The Sprague–Dawley rats were purchased from Beijing Weitonglihua Laboratory Animal, Company Limited. The animals were maintained in the animal facility at the Xinxiang Medical University and family- or pair-housed in a temperature-controlled animal room with 12/12-h light/dark cycle. Food and water were available *ad libitum*. All animal experiments were performed in accordance with the protocols approved by the Institutional Animal Care and Use Committee of Xinxiang Medical University.

### Reagents

All chemicals were purchased from Sigma company unless otherwise stated. The antibodies to Cdk5 (C8 and DC17) and p35 (C19) were purchased from Santa Cruz Biotechnology, Santa Cruz, CA, USA. Phospho-histone H1 (32078) was purchased from Upstate. The phospho-tau (Ser202; 11834), phospho-tau (Ser404; 11837), total tau (4019), β-actin (4970), and horseradish peroxidase-conjugated goat anti-mouse (7076) and anti-rabbit (7074) IgG antibodies were from Cell Signaling Technology, Danvers, MA, USA. The lentiviral particles encoding enhanced green fluorescent protein (GFP) were from Santa Cruz Biotechnology, Santa Cruz, CA, USA.

### Primary Neuron Culture

Cultured hippocampal neurons were prepared from rat embryos at day E18, seeded on 60-mm plates coated with poly-D-lysine (Sigma–Aldrich, St. Louis, MO, USA, P1149, 5 μg/ml), recovered in the plating media (Dulbecco’s modified eagle medium with 10% fetal bovine serum, 2 mM L-glutamine, and 0.5% glucose) for 4 h, and then maintained in neurobasal medium supplemented with 2% B27 and 0.5 mM L-glutamine before use. To examine Aβ-induced p25/p35 changes, the hippocampal neurons were treated with 500 nM Aβ for 2 h at DIV10 with or without metformin pretreatment and then the cells were harvested, lysed, and subjected to western blot analysis using the indicated antibodies.

### Cdk5 *in vitro* Kinase Assay and Western Blotting

The Cdk5 kinase activity was measured using a previously established Cdk5 kinase activity assay (Zhang et al., 2010, 2015; Qu et al., 2011; Sheng et al., 2016). Briefly, mouse hippocampi were homogenized and lysed, and then Cdk5/p35/p25 protein complex was co-immunoprecipitated using Cdk5 antibody (C8, 1 ug/500 g protein lysate) overnight and pulled down by protein-A/G agarose beads at 4°C for 60 min. The beads were then washed three times with lysis buffer and once with kinase reaction buffer. The kinase assay was then performed with 10 μg histone H1 and magnesium/ATP mixture at 30°C for 30 min, followed by western blotting using the phospho-histone H1 antibody. Cdk5, p35, phosphor-tau (p-S202), phosphor-tau (p-S404), tau, and β-actin were blotted using anti-Cdk5 (1:1,000; Santa Cruz Biotechnology, Santa Cruz, CA, USA), anti-p35 (1:2,000; Santa Cruz Biotechnology, Santa Cruz, CA, USA), anti-phospho-tau (1:2,000; Cell Signaling Technology, Danvers, MA, USA), anti-tau (1:2,000; Cell Signaling Technology, Danvers, MA, USA), and anti-actin (1:5,000; Cell Signaling Technology, Danvers, MA, USA). The band intensity was quantified by chemiluminescence and densitometric scanning of the films under linear exposure conditions using ImageJ software (NIH).

### Virus Infection, Dendritic Spine Quantification, Hippocampal Slice Preparation, and Electrophysiology

For *in vivo* lentivirus expression, ~5-month-old animals were initially anesthetized by an intraperitoneal injection of chloral hydrate and then placed in a stereotaxic frame. Lentiviral particles with pLenti-hSynapsin-GFP were delivered into the mouse hippocampal CA1 region (AP: −2.00 mm, ML: ±1.70 mm, DV: −1.4 mm, relative to the Bregma) for 4 weeks with an injection volume of 50 nl viral solution using a glass pipette. After surgery, the animals were placed under a heating lamp until reawakening. At 4 weeks later, the animals were sacrificed, and coronal slices with 50-μm thickness were prepared using Leica Vibratome VT1000S. For imaging acquisition of dendritic spines in GFP-labeled CA1 hippocampal pyramidal neurons, the images were obtained with an Olympus Fluoview FV1000 confocal microscope with a 60× oil-immersion objective using z-stack scanning mode, and then image analysis was performed with Metamorph software. For dendritic spine density quantification, three dendrite segments of secondary apical dendrites from each neuron were analyzed. The acute mouse hippocampal slices were from 6-month-old male and female mice. The animals were deeply anesthetized by chloral hydrate and decapitated. The brain containing the hippocampus was quickly removed and placed into a cold (0°C), oxygenated physiological solution containing 125 mM NaCl, 2.5 mM KCl, 1.25 mM NaH_2_PO_4_, 25 mM NaHCO_3_, 1 mM MgCl_2_, 25 mM dextrose, and 2 mM CaCl_2_ (pH 7.4). Then, parasagittal slices with 350–400-μm thickness were cut from the brain blocks. These slices were kept at 37.0 ± 0.5°C in an oxygenated physiological solution for ~0.5 h before experiments. Then, the brain slices were kept at room temperature for Aβ treatment experiments and electrophysiology recording. Field excitatory postsynaptic potentials (fEPSP) were recorded in the CA1 stratum radiatum region. Long-term potentiation (LTP) was induced with a conditioning stimulus consisting of three theta burst trains with 60-s intervals. Each theta burst train itself consisted of 10 5-Hz series of four 100-Hz pulses. fEPSP slopes were measured to reflect the synaptic activity and the slopes acquired during the last 10 min were analyzed by MED64 Mobius. A statistical analysis was performed with GraphPad Prism8.0.

### Surface Biotinylation Assay

Acute mouse hippocampal slices were prepared from 6-month-old mice and recovered for 0.5 h at 37.0 ± 0.5°C in oxygenated physiological solution. Then, the slices were washed twice with ice-cold phosphate buffer and subsequently incubated with 0.5 mg ml^−1^ EZ-link-sulfo-SS-Biotin (Pierce) in phosphate buffer for 30 min. The biotin solution was then removed, and the remaining biotin was quenched by 50 mM Tris solution (pH 7.4) for 2 min. The slices were then washed three times with ice-cold phosphate buffer and lysed with the radioimmunoprecipitation assay buffer supplemented with protease inhibitor cocktail. The biotinylated proteins were immunoprecipitated with streptavidin agarose A/G for 1–2 h at 4°C. The protein A/G beads were washed three times with lysis buffer, after which 2× SDS sample buffer was added to elute the biotinylated proteins for western blot analysis.

### Behavioral Test: Morris Water Maze

The Morris Water Maze (MWM) test was performed as described before. Briefly, chronic metformin (met, 200 mg per kilogram of body weight) or saline (sal) was administrated into the mice 5 days before training, and this administration lasted throughout the whole testing period of 10 days. The circular water maze pool was 120 cm and the platform was 10 cm in diameter. The water was maintained at room temperature (22–23°C) and made opaque by the addition of white tempera to enhance contrast. Male mice (aged ~6 months old, ~10–12 mice per group) were handled daily for 5 days before the start of the experiment. During the experiment, the mice were trained three times per day with 30-min interval over five consecutive days (days 1–5). In each trial, the training lasted 90 s or until the mouse found the platform. If the mouse did not find the platform in 90 s, it was guided to the platform and made to stay there for 10 s before being returned to the home cage. Probe test was then performed on day 6. The mouse was put into the tank to swim for 60 s without the platform, and its performance was assessed by quantifying the time spent in the target quadrant in which the hidden platform was originally placed. The animal behavior was recorded with a video camera and analyzed by Ethovision XT 7.0 (Noldus).

### Statistical Analysis

Statistical results were reported as mean ± SEM from at least six independent experiments. The statistical significance of the means (*p* < 0.05; two sides) was determined by two-way ANOVA with Tukey’s *post hoc* test.

## Results

### Metformin Administration Inhibits Cdk5 Kinase Activity

**Figure 1:**
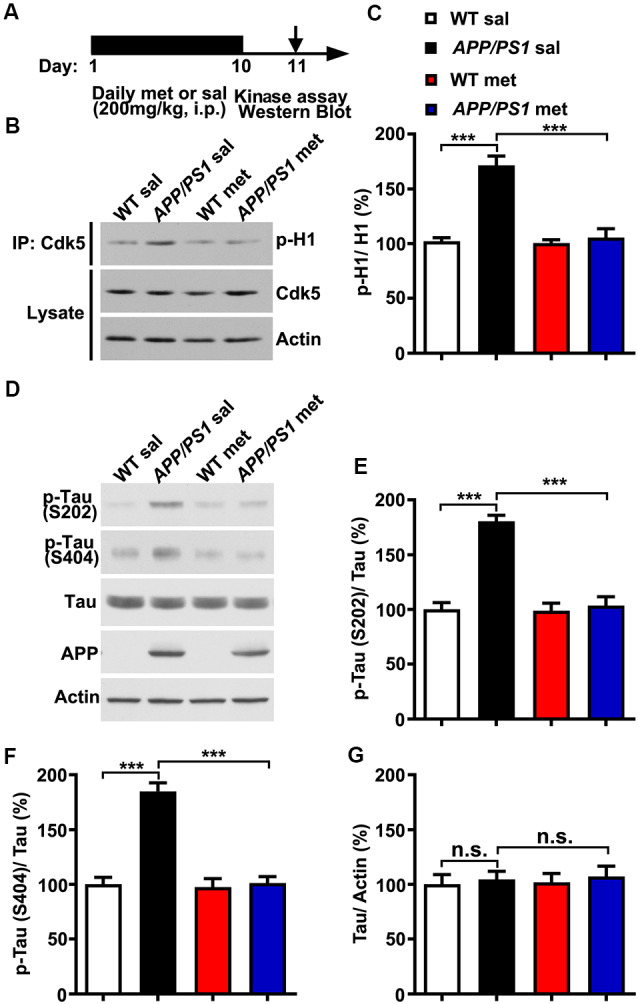
Chronic metformin treatment prevents Cdk5 hyper-activation in *APP/PS1* mutant mice. **(A)** Schematic diagram showing the experimental design. Metformin (met) or saline (sal) was intraperitoneally (i.p.) injected over 10 days, followed by *in vitro* kinase assay and western blot analysis. **(B)** Representative western blots of hippocampal lysates from sal- and met-treated WT and *APP/PS1* mice to measure the Cdk5 activity. **(C)** Quantification of the phosphorylation level of histone H1 (p-H1; mean ± SEM, *n* = 8 hippocampi from eight mice; ***p < 0.001, two-way ANOVA with Tukey’s *post hoc* test). **(D)** Representative western blots of hippocampal lysates from sal- and met-treated WT and *APP/PS1* mice. **(E)** Quantification of the phosphorylation level of tau at serine 202 (S202; mean ± SEM, *n* = 8 hippocampi from eight mice; ***p < 0.001, two-way ANOVA with Tukey’s *post hoc* test). **(F)** Quantification of the phosphorylation level of tau at serine 404 (S404; mean ± SEM, *n* = 8 hippocampi from eight mice; ***p < 0.001, two-way ANOVA with Tukey’s *post hoc* test). **(G)** Quantification of the protein level of total tau (mean ± SEM, *n* = 8 hippocampi from eight mice; two-way ANOVA with Tukey’s *post hoc* test). n.s., not significant.

To examine whether and how metformin had beneficial therapeutic effects in *APP/PS1* mice, adult (~6 month) wild-type (WT) and *APP/PS1* mice were injected intraperitoneally (i.p.) with metformin (met; 200 mg/kg/day) or saline (sal) for 10 days. Mouse hippocampal lysate was then subjected to *in vitro* Cdk5 kinase assay and western blot analysis 24 h after the last metformin administration (Figure 1A). Consistent with previous studies which showed that Cdk5 signaling pathway is hyper-activated in *APP/PS1* mice (Qu et al., 2011; Fu et al., 2014; Chen et al., 2015), Cdk5 activity was elevated in the adult *APP/PS1* mouse hippocampus. More interestingly, metformin treatment for 10 days prevented Cdk5 hyper-activation as revealed by the reduced phosphorylation level of histone H1 in the *APP/PS1* mouse hippocampus (Figures 1B,C), with no changes in the Cdk5 protein level. Moreover, the Cdk5-dependent phosphorylation levels of tau at serine 202 (S202) and serine 404 (S404) were also elevated in *APP/PS1* mice, whereas metformin treatment for 10 days reduced the levels of phosphorylated tau at S202 and S404 in the hippocampus of *APP/PS1* mice to WT level without any changes in the total tau protein level (Figures 1D–G). These data consistently implicated that Cdk5 signaling pathway was hyper-activated in *APP/PS1* mice and chronic metformin administration suppressed Cdk5 hyper-activation and restored its dependent phosphorylation of tau to a normal level.

### Metformin Treatment Inhibits Cleavage of p35 Into p25

**Figure 2:**
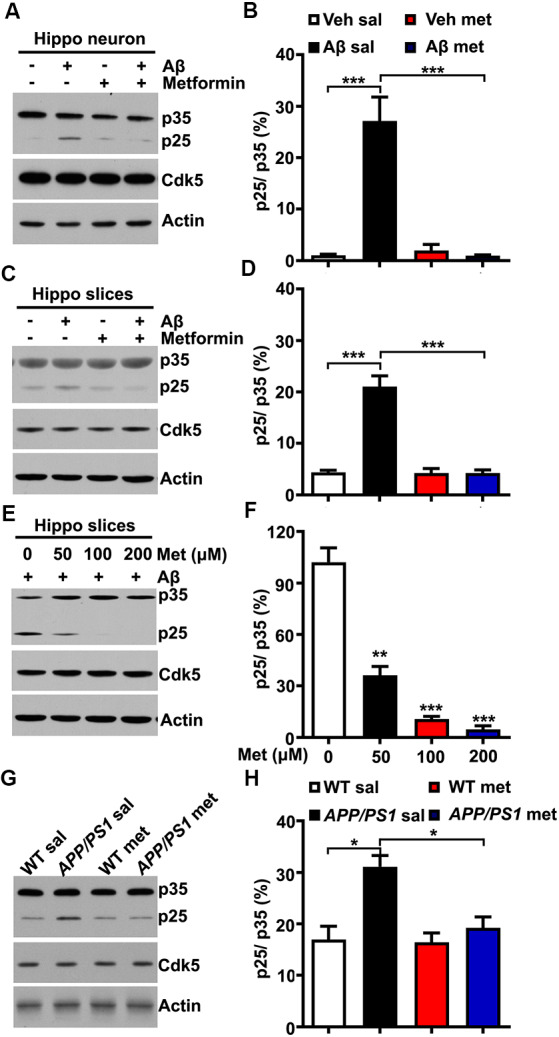
Metformin treatment inhibits p35 cleavage into p25. **(A)** Representative immunoblots of cultured hippocampal neuron lysates from vehicle- and Aβ (500 nM)-treated neurons with or without metformin (100 μM) pretreatment. **(B)** Quantification of p25 to p35 ratio (mean ± SEM, *n* = 8 from four independent experiments; ***p < 0.001, two-way ANOVA with Tukey’s *post hoc* test). **(C)** Representative immunoblots of lysates from vehicle- and Aβ (500 nM)-treated hippocampal slices with or without metformin (100 μM) pretreatment. **(D)** Quantification of p25 to p35 ratio (mean ± SEM, *n* = 8 from four independent experiments; ***p < 0.001, two-way ANOVA with Tukey’s *post hoc* test). **(E)** Representative immunoblots of lysates from Aβ (500 nM)-treated hippocampal slices with different dosages of metformin (0–200 μM) pretreatment. **(F)** Quantification of p25 to p35 ratio (mean ± SEM, *n* = 8 from four independent experiments; **p < 0.01, ***p < 0.001, two-way ANOVA with Tukey’s *post hoc* test). **(G)** Representative western blots of hippocampal lysates from sal- and met-treated WT and *APP/PS1* mice. **(H)** Quantification of p25 to p35 ratio (mean ± SEM, *n* = 8 from four independent experiments; *p < 0.05, two-way ANOVA with Tukey’s *post hoc* test).

The underlying mechanism by which metformin administration could block Cdk5 hyper-activation in *APP/PS1* mice is not clear. Several previous studies reported that Cdk5 activator p35 was cleaved into p25 in a calpain-dependent manner, resulting in elevated Cdk5 activation and tau hyper-phosphorylation in AD patient brains and mouse models of AD when the intracellular calcium concentration was abnormally high (Kusakawa et al., 2000; Lee et al., 2000). Meanwhile, a recent study reported that metformin treatment protected the neurons from quinolinic acid-induced neurotoxicity by inhibiting the intracellular calcium increase (Jang and Park, 2018). A plausible explanation is that the effect of metformin treatment on the change of Cdk5 signaling is due to the reduced conversion of p35 to p25. To test this hypothesis, cultured hippocampal neurons were treated with Aβ oligomers (500 nM for 2 h) to mimic neurotoxicity. In agreement with previous findings (Lee et al., 2000), the application of Aβ oligomers in cultured hippocampal neurons induced p35 cleavage to p25 as evidenced by the increased p25/p35 ratio, whereas pretreatment with metformin (100 μM) effectively inhibited the Aβ-induced cleavage of p35 (Figures 2A,B). To independently confirm these data, acute hippocampal slices from adult (~6 months old) WT mice were also treated with Aβ oligomers in the presence or absence of metformin pretreatment. Consistent with the data obtained in cultured hippocampal neurons, metformin effectively blocked the Aβ oligomer-induced cleavage of p35 into p25 (Figures 2C,D). To further investigate how metformin treatment affects p35 cleavage, acute hippocampal slices were treated with Aβ oligomers in the presence of metformin at different concentrations. Interestingly, metformin treatment prevented p35 cleavage into p25 in a dose-dependent manner (Figures 2E,F), suggesting that metformin directly impinged on p35/p25 cleavage. Furthermore, an increased proteolytic cleavage of p35 to p25 was also detected in the *APP/PS1* mouse hippocampus, and chronic metformin administration for 10 days restored the p25/p35 ratio to WT level (Figures 2G,H). Collectively, these data suggested an interesting molecular mechanism by which metformin treatment prevented the hyper-activation of Cdk5 signaling in the *APP/PS1* mouse hippocampus by blocking p25 overproduction.

### Chronic Metformin Treatment Rescues Dendritic Spine Loss and Surface AMPA Reduction in *APP/PS1* Mice

**Figure 3:**
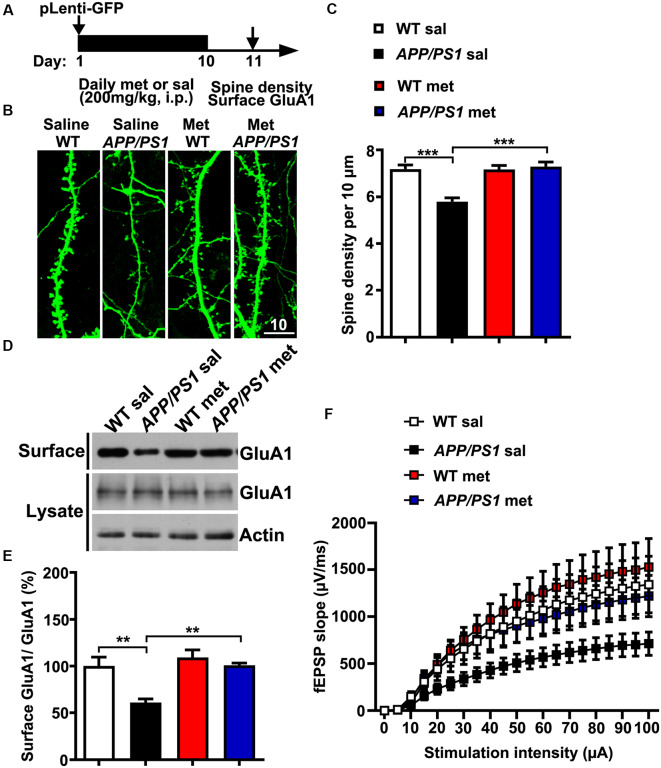
Chronic metformin treatment corrects dendritic spine loss and surface GluA1 reduction in *APP/PS1* mutant mice. **(A)** Schematic diagram showing the experimental design. Metformin (met) or saline (sal) was i.p. injected over 10 days, followed by dendritic spine density analysis and surface GluA1 labeling. For the dendritic spine analysis, lentivirus encoding enhanced green fluorescent protein (EGFP) was injected into the hippocampal CA1 region on D 1. (**B**) EGFP-labeled CA1 dendritic spines from sal- and met-treated WT and *APP/PS1* mice. Scale bar, 10 μm. **(C)** Quantification of spine density in CA1 pyramidal neurons, measured as the spine number per 10 μm of dendrite length (mean ± SEM, *n* = 36 dendrites from 12 CA1 pyramidal neurons, four independent experiments; ***p < 0.001, two-way ANOVA with Tukey’s *post hoc* test). **(D)** Representative immunoblots of surface GluA1 and total GluA1 from sal- and met-treated WT and *APP/PS1* mice. **(E)** Quantification of surface GluA1 to total GluA1 ratio (mean ± SEM, *n* = 8 mice from four independent experiments; **p < 0.01, two-way ANOVA with Tukey’s *post hoc* test). **(F)** The input–output (I/O) curves in response to stimulus in the mouse hippocampus CA1 region in WT and *APP/PS1* mice administrated with saline or metformin. The I/O curve was measured by averaging the field excitatory postsynaptic potential slopes against different stimulus intensities from 5 to 100 μA (*n* = 10–12 slices from six to eight mice).

Neurons from individuals with AD and mouse models of AD exhibit reduced dendritic spine density and comprised excitatory glutamatergic neurotransmission (Qu et al., 2011; Sheng et al., 2016; Bai et al., 2017). To further examine whether metformin had some beneficial effects on synaptic dysfunctions in *APP/PS1* mice, adult (~6 months old) WT and *APP/PS1* mice were injected intraperitoneally (i.p.) with met (200 mg/kg per day) or sal for 10 days (Gantois et al., 2017). The mice were then subjected to spine morphogenesis analysis and surface AMPA labeling experiments 24 h after the last metformin administration (Figure 3A). The *APP/PS1* mice displayed spine loss as evidenced by decreased spine density from CA1 pyramidal neurons. Metformin administration for 10 days corrected the dendritic spine density to WT level, implicating a protective effect of metformin on spine abnormalities (Figures 3B,C). In addition, compromised surface AMPA trafficking was also observed in *APP/PS1* mouse hippocampus as revealed by the reduced surface expression of AMPA subunit GluA1. More importantly, metformin treatment rescued the surface GluA1 expression to a normal level (Figures 3D,E). Furthermore, adult *APP/PS1* mice treated with saline displayed a significantly reduced CA3–CA1 synaptic transmission strength as indicated by an obvious decrease in the fEPSP input–output (I/O) relationship. The chronic administration of metformin reversed this neurotransmission defect (Figure 3F). Altogether the chronic application of metformin for 10 days rescued the various synaptic abnormalities including spine loss, surface GluA1 expression reduction, and decrease in basal synaptic transmission in the hippocampus of *APP/PS1* mice.

### Chronic Administration of Metformin Rescues LTP Defects and Spatial Memory Deficits in *APP/PS1* Mice

**Figure 4:**
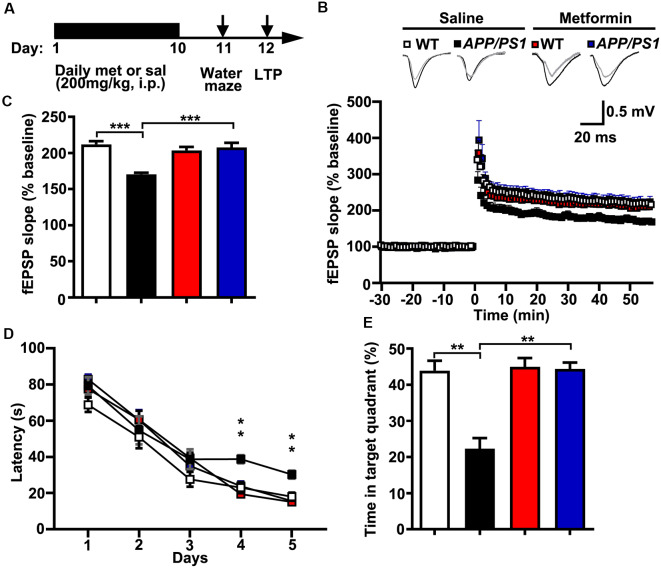
Chronic metformin treatment reverses long-term potentiation (LTP) defect and corrects spatial memory deficit in *APP/PS1* mutant mice. **(A)** Schematic diagram shows the experimental design. Metformin (met) or saline (sal) was i.p. injected over 10 days, followed by an analysis of spatial memory and LTP. **(B)** LTP at the CA3–CA1 synapses was measured in acute slices prepared from saline- and metformin-treated WT and *APP/PS1* mice. **(C)** Quantification of field excitatory postsynaptic potential slope during the last 10 min of recording (WT sal: 2.12 ± 0.04, *APP/PS1* sal: 1.71 ± 0.02, WT met: 2.04 ± 0.05, *APP/PS1* met: 2.08 ± 0.07, mean ± SEM, *n* = 12–16 slices from 6 to 8 mice; ***p < 0.001, two-way ANOVA with Tukey’s *post hoc* test). **(D)** The escape latency was measured from saline- and metformin-treated WT and *APP/PS1* mice along 5 days of testing. Note that the administration of *APP/PS1* mice with metformin reduced the escape latency at D 4 and D 5. **(E)** Quantification of the time spent in the target quadrant from sal- and met-treated WT and *APP/PS1* mice in the probe trial test. Note that the administration of *APP/PS1* mutant mice with metformin increased the time spent in the target quadrant (mean ± SEM, *n* = 12 mice; **p < 0.01, two-way ANOVA with Tukey’s *post hoc* test).

The LTP of synaptic transmission is critical for hippocampus-dependent learning and memory. It is widely accepted that a hallmark of individuals with AD or AD mouse models is impaired LTP and its associated spatial memory (Fu et al., 2014). Given these data that metformin treatment reversed the hyper-activation of Cdk5 signaling and rescued synaptic dysfunctions, it was of great interest to examine whether metformin treatment improved the LTP expression and the spatial memory in *APP/PS1* mice (Figure 4A). Thus, LTP at the hippocampal CA3–CA1 synapses was examined. Theta burst stimulation-induced CA3–CA1 LTP was impaired in acutely prepared hippocampal slices from *APP/PS1* mice, whereas chronic treatment with metformin for 10 days rescued the LTP impairment (Figures 4B,C). Moreover, the effect of chronic metformin administration on spatial learning and memory was examined in *APP/PS1* mice. Adult (~6 months old) *APP/PS1* mice and their littermate controls were administrated daily with metformin for 10 consecutive days, and then the spatial memory was examined using the MWM behavioral test (Figure 4A). These mice were trained for 5 days, and the escape latency to get to the hidden platform was recorded. No significant difference in the escape latency was found from D 1 to D 3 in both saline- and metformin-treated WT and *APP/PS1* mice, indicating that these mice showed no defects in motor coordination and swimming abilities. At D 4 and D 5, the *APP/PS1* mice treated with saline showed a much higher escape latency, whereas the *APP/PS1* mice treated with metformin significantly improved their performance, while the WT mice treated with metformin did not change their performance obviously compared to the WT mice treated with saline (Figure 4D). At 24 h after the last training day, the hidden platform was removed and a probe trial was performed, and the time mice spent in the target quadrant was measured as a spatial memory index. In the probe test, the WT mice treated with saline showed much more time in the target quadrant (~44%, Figure 4E), while the *APP/PS1* mice treated with saline showed no quadrant preference (~22%, Figure 4E), indicating an impaired spatial memory in *APP/PS1* mice. However, the *APP/PS1* mice with chronic metformin application significantly increased their time spent in the target quadrant to a level comparable to that of the WT mice (~44%, Figure 4E), suggesting that metformin treatment significantly improved the spatial memory in *APP/PS1* mice.

## Discussion

In the current study, we combined pharmacological, molecular and cellular, electrophysiological, and behavioral techniques to study the roles of Cdk5 signaling in AD pathogenesis and how chronic metformin treatment had beneficial effects on the synaptic malfunctions and the cognitive defects in the *APP/PS1* mice through the regulation of the Cdk5 signaling pathway. We firstly confirmed that Cdk5 was hyper-activated in the *APP/PS1* mouse hippocampus. Then, we showed that the widely used anti-diabetes drug, metformin, could inhibit Cdk5 activity by preventing p35 cleavage into p25. Furthermore, the blockade of Cdk5 activity by chronic metformin administration could restore spine loss, reduced surface GluA1 trafficking, impaired synaptic plasticity, and defective spatial memory to those of WT level in the *APP/PS1* mice, unveiling an unanticipated role of metformin in alleviating AD progression. Our results indicated that the widely used type 2 diabetes drug, metformin, could be promptly proposed as a promising drug for patients with AD.

At present, there is no cure for AD, and several recent completed clinical trials targeting Aβ deposits either failed or were not promising (Hung and Fu, 2017). Thus, re-examining the molecular and the cellular mechanisms underlying AD pathogenesis may provide us with new insights and present some new potential therapeutic targets to cure AD. Indeed numerous pioneering studies have demonstrated that Cdk5 plays an indispensable role in synaptic plasticity and its deregulation is capable of initiating the early Alzheimer’s synaptic pathology, further resulting in neuronal network dysfunction and cognitive impairment and decline in AD (Qu et al., 2011; Sheng et al., 2016). Therefore, there is a good reason to propose that Cdk5 inhibition is a promising strategy to intervene on the early pathogenesis of this disorder. For example, Cdk5 inhibitor roscovitine and small peptides that can disrupt Cdk5/p35/p25 interaction have been used for AD intervention (Sundaram et al., 2013; Tell and Hilgeroth, 2013; Shukla et al., 2017). Meanwhile, accumulating studies showed that neurons and pancreatic cells share many very important signaling pathways. For instance, Cdk5 signaling is reported to be dysregulated in both diabetes and AD patients and Cdk5 hyper-activation is also sufficient to lead to the pathogenesis of diabetes (Lalioti et al., 2009; Mora and Aguanno, 2018). In addition, clinical studies revealed a highly positive correlation between diabetes onset and AD progression (Akter et al., 2011; Sluggett et al., 2020). Altogether, we are eager to examine if the anti-diabetes drug, metformin, could be a potential AD treatment drug. Intriguingly, here we found that the anti-diabetes drug, metformin, could inhibit Cdk5 activity, and we identified a novel mechanism that metformin inhibited Cdk5 activity by preventing the calpain-dependent cleavage of p35 to p25. More importantly, chronic metformin administration in *APP/PS1* mice rescued synaptic failures and improved learning and memory. It is noteworthy that, consistent with our current study, Chen et al. (2015) reported that another anti-diabetes drug, pioglitazone, could also alleviate AD pathogenesis by promoting p35 degradation in AD mouse models. Although the underlying mechanism is different, both studies showed that anti-diabetes drugs could be a new potential solution to treat AD patients. However, it remains unclear how metformin and pioglitazone treatment inhibited Cdk5 activity with two different mechanisms. In this regard, it will be interesting to figure out the underlying mechanism by which metformin decreases p35 cleavage and pioglitazone promotes p35 degradation. Moreover, several other studies also reported that metformin showed promising effects for alleviating the symptoms of AD and could be a potential therapeutic drug for neurodegenerative diseases (Ou et al., 2018; Rotermund et al., 2018; Farr et al., 2019). For example, Ou et al. (2018) reported that metformin treatment prevented amyloid plaque deposition and memory impairment in *APP/PS1* mice by enhancing the AMPK activation. Meanwhile, another study reported that metformin ameliorated the core deficits of fragile X syndrome by AMPK activation (Gantois et al., 2017). Thus, it will be interesting to check if there is any crosstalk between Cdk5 and AMPK pathway in alleviating AD symptoms after metformin treatment.

Another interesting question is how metformin could exert protective effects on dendritic spine density, surface GluA1 expression, LTP, and its associated learning and memory in AD mouse models by suppressing the Cdk5 hyper-activation. Under normal conditions, Cdk5 activity is precisely regulated and highly suppressed in adult brains to maintain synaptic integrity. However, under pathological conditions, Cdk5 is hyper-activated when the neurons are exposed to cellular stresses such as Aβ oligomers and calcium influx. The Cdk5 hyper-activation will impair synaptic strength structurally and functionally *via* the inhibitory phosphorylation of its synaptic substrates. For example, the Cdk5 substrate WAVE1 is important for dendritic spine growth and maturation and the Cdk5-dependent phosphorylation inhibits its activity and decreases spine density and maturation (Kim et al., 2006; Sung et al., 2008). In parallel, Cdk5 negatively regulates N-methyl-D-aspartate receptor-mediated synaptic transmission through direct suppression of the surface expression of NR2B (Zhang et al., 2008; Plattner et al., 2014). Moreover, the PKA-dependent phosphorylation of GluA1 and GluN1 drives their surface expression, while the Cdk5-dependent phosphorylation of DARPP-32 inhibits PKA activity, which in turn reduces the phosphorylation levels and the surface expressions of GluA1 and GluN1 (Bibb et al., 1999; Zhang et al., 2015). Therefore, when p35 cleavage into p25 is inhibited in response to chronic metformin administration, the Cdk5 hyper-activation is suppressed and the “brake” that restrains the synaptic strength is released. As a result, metformin restores dendritic spine density and maturation as well as surface GluA1 expression to a normal level, further leading to improved LTP expression and spatial memory in *APP/PS1* mice. Thus, we proposed that metformin exerted its protective effects in *APP/PS1* mice by preventing the Cdk5 hyper-activation and its phosphorylation of synaptic substrates.

Furthermore, it is well established that neurodegenerative diseases, psychiatric disorders, and diabetes seem to share many physiological characteristics. Given that there are many widely used FDA-approved drugs to treat diabetes, it is of great interest to examine if these drugs can be used to cure some neurodegenerative diseases or psychiatric disorders. Indeed Zemdegs et al. (2019) reported that metformin treatment could reduce the anxiety symptoms in mice. In addition, studies have shown that the incretin hormone, glucagon-like peptide-1, which has antidiabetic properties, can play a neuroprotective role in the brain and has demonstrated promising effects in animal models of AD and PD (Hölscher, 2014; Batista et al., 2019). Collectively, all these data raise a very interesting possibility that the anti-diabetes drugs can be potential promising drugs to cure neurodegenerative diseases and psychiatric disorders.

## Data Availability Statement

The datasets generated for this study are available on request to the corresponding author.

## Ethics Statement

The animal study was reviewed and approved by Xinxiang Medical University.

## Author Contributions

This study was initiated and designed by JW. YW, F-LG, XG, and XX performed the brain slice electrophysiology recordings and analyzed the data. JZ, SL, and XY performed the primary neuron culture, and XFY, LZ, YY, and LF performed the Morris Water Maze (MWM). JZ and YW performed the western blotting and the surface biotinylation assay. JW and YW wrote the manuscript.

## Conflict of Interest

The authors declare that the research was conducted in the absence of any commercial or financial relationships that could be construed as a potential conflict of interest.
